# Convergent microRNA actions coordinate neocortical development

**DOI:** 10.1007/s00018-014-1576-5

**Published:** 2014-02-12

**Authors:** Olga Barca-Mayo, Davide De Pietri Tonelli

**Affiliations:** Department of Neuroscience and Brain Technologies, Istituto Italiano di Tecnologia, Via Morego 30, 16163 Genoa, Italy

**Keywords:** Rodents, Cerebral cortex, Development, MicroRNAs

## Abstract

Neocortical development is a complex process that, at the cellular level, involves tight control of self-renewal, cell fate commitment, survival, differentiation and delamination/migration. These processes require, at the molecular level, the precise regulation of intrinsic signaling pathways and extrinsic factors with coordinated action in a spatially and temporally specific manner. Transcriptional regulation plays an important role during corticogenesis; however, microRNAs (miRNAs) are emerging as important post-transcriptional regulators of various aspects of central nervous system development. miRNAs are a class of small, single-stranded noncoding RNA molecules that control the expression of the majority of protein coding genes (i.e., targets). How do different miRNAs achieve precise control of gene networks during neocortical development? Here, we critically review all the miRNA–target interactions validated in vivo, with relevance to the generation and migration of pyramidal-projection glutamatergic neurons, and for the initial formation of cortical layers in the embryonic development of rodent neocortex. In particular, we focus on convergent miRNA actions, which are still a poorly understood layer of complexity in miRNA signaling, but potentially one of the keys to disclosing how miRNAs achieve the precise coordination of complex biological processes such as neocortical development.

## Introduction

The structure of the mammalian neocortex, the site where higher cognitive behaviors are generated, is organized in six layers and is composed of different neuronal subtypes and glia. Pyramidal-projection glutamatergic neurons are the predominant type of cortical neurons, accounting for 75–85 % of the total neuronal population, depending on the species. The remaining 15–25 % of cortical neurons are GABAergic interneurons [1–3].

**Fig. 1:**
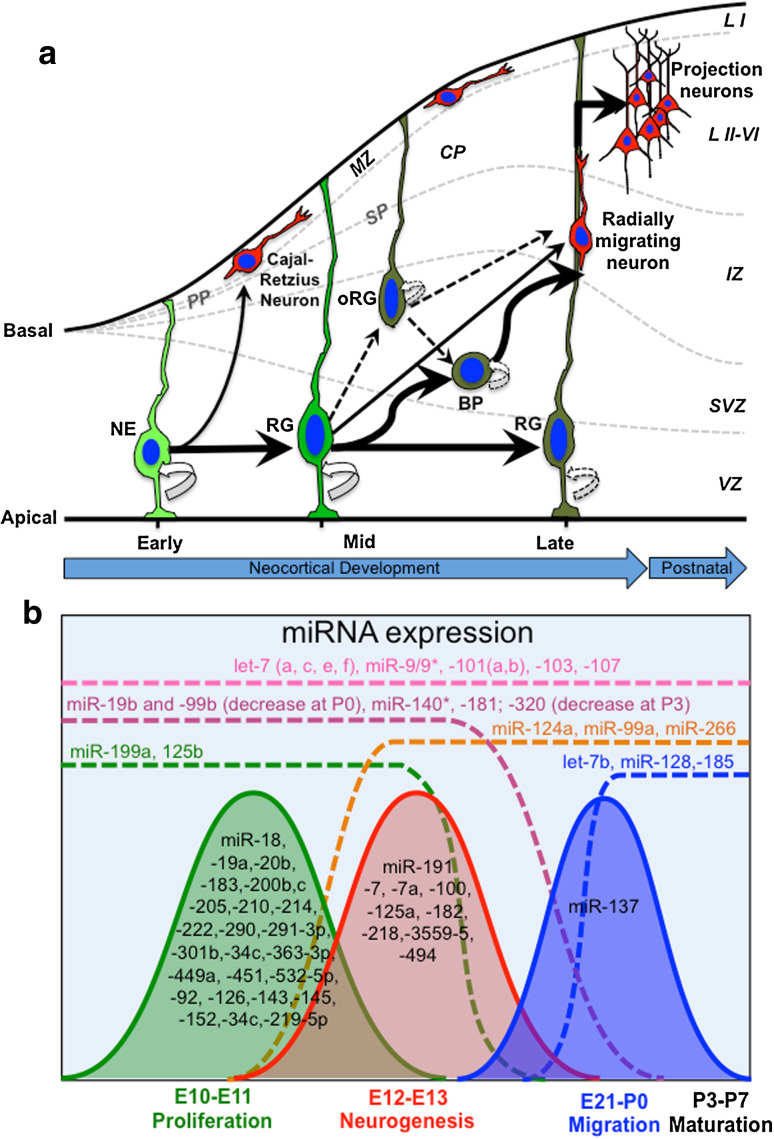
Cell biology and miRNA expression in developing rodent neocortex. **a** In the early phase of cortical development, the telencephalon is composed of single layer neuroepithelial (NE) cells that divide at the ventricular (apical) surface of the ventricular zone (VZ). In this phase, NE cells undergo a massive expansion, mostly by symmetric self-amplifying divisions (*curved arrow*). At the onset of neurogenesis, NE cells progressively start to divide asymmetrically. NE cells can generate the Cajal-Retzius neurons, which migrate out from the VZ toward the basal lamina (basal) and form the preplate (PP). During development, the PP is split into the marginal zone (MZ) and subplate (SP) by incoming neurons, giving rise to the cortical plate (CP). During the mid phase of cortical development, NE cells give rise to additional and fate-restricted subtypes of neural progenitors cells (NPCs). NPCs deriving from NE cells include radial glia (RG) and short neural precursors (not shown) collectively referred to as apical NPCs, and progenitors that delaminate from the VZ and divide in the subventricular zone (SVZ), basal progenitors (BP), and outer radial glia (oRG) (collectively referred to as basal NPCs). Apical and basal NPCs can generate progenitors, neurons, or both, and undergo a limited number of symmetric self-amplifying divisions (*dashed curved arrows*). During mid and late phases of cortical development, several types of glutamatergic cortical projection neurons are generated by apical or basal NPCs. Newborn glutamatergic cortical neurons migrate radially from the VZ/SVZ, and by somatic translocation along the basal processes of RG cells, cross the intermediate zone (IZ) and accumulate into the CP in an inside-out way. The early-born neurons form the postnatal cortical layers VI–V (L VI–V) (deep layers), while later-born neurons accumulate above the deep layers, forming the cortical layers IV–II (superficial layers, L III–II). Layer I (L I) originates from the MZ. **b** microRNAs (miRNAs) show dynamic patterns of expression in developing rat cortex. The expression pattern of miRNAs in developing rat cortex can be roughly classified into four main trends: (1) miRNAs that are continuously expressed throughout development (*pink dashed line*); (2) miRNAs that start to be expressed during early development and decline in their expression during development, or perinatally (*purple* and *green dashed lines*); (3) miRNAs that start to be expressed in mid or late development and then also remain expressed in postnatal brain (*orange* and *blue dashed lines*); and (4) miRNAs that are specifically expressed during a short period during development (*curved lines*). Co-expressed miRNAs might cooperate to modulate the activities of transcription factors and signaling networks, which are required during specific phases of cortical development

The neocortex is generated by the development of the foremost region of the neural tube: the telencephalon. This region comprises the dorsal telencephalon (generating almost exclusively excitatory glutamatergic projection neurons) and the ventral telencephalon (producing inhibitory GABAergic interneurons). Pyramidal neurons of the dorsaltelencephalon are born locally in the germinal zones [i.e., the ventricular (VZ) and subventricular zone (SVZ)], migrate toward the cortical plate by somal translocation along radial glia (RG) (Fig. 1a), and assemble in an inside-out manner to establish the six layers that characterize the laminar structure of the neocortex [4]. In contrast, GABAergic interneurons arise from the germinal zones of the ventral telencephalon and make long journeys, following tangential migratory routes, to their final destinations in the neocortex [4]. Finally, but at a later developmental time compared to cortical neurons, germinal zones give rise to the two major glial cell types of the neocortex, namely, astrocytes and oligodendrocytes [5]. Thus, neocortical development (or corticogenesis) is a complex neurodevelopmental process that requires the precise coordination of cell proliferation, differentiation, as well as subtype specification and migration.

How are the pyramidal-projection glutamatergic neurons generated during neocortical development? Several years of study have contributed to clarifying the transcriptional mechanisms that control the generation of cortical neurons during development [6, 7], but in recent years, novel important mechanisms of gene regulation have been discovered. Examples of these mechanisms include epigenetics [8] and noncoding RNAs [9]. microRNAs (miRNAs) are a class of small, single-stranded noncoding RNA molecules that control the expression of the majority of protein coding genes (i.e., targets), mainly at the post-transcriptional level [10]. miRNAs and their binding sites in target messenger RNAs (mRNAs) are often evolutionarily conserved among distant organisms [11, 12]. Moreover, miRNAs that are expressed in the developing central nervous system (CNS) exert a prominent role as regulatory molecules that coordinate gene networks during neocortical development, as well as in brain function and dysfunction [13–18]. How do the different miRNAs achieve precise control of gene networks during neocortical development? Here, we critically review all the miRNA-target interactions validated in vivo, with relevance to the generation and migration of pyramidal-projection glutamatergic neurons and initial formation of cortical layers in the embryonic development of the neocortex in mice and rats (hereinafter collectively referred to as rodents). In particular, we first describe the miRNA biogenesis pathway, and then the diversity of the neural stem and progenitor cells (NSCs/NPCs) that give rise to pyramidal-projection glutamatergic neurons. Next, we describe the pattern of miRNA expression, and review the studies that have used the genetic inhibition of miRNA biogenesis as a strategy to investigate the global role of miRNAs in the context of neocortical development. Finally, we review the studies in which manipulation of specific miRNAs have been used as a strategy to identify targets and mechanisms that control neocortical development in vivo. Moreover, here we focus on convergent miRNA action on targets, which is still a poorly understood layer of complexity in miRNA signaling, but potentially one of the keys to disclosing how miRNAs achieve the precise control of molecular programs, pathways and biological functions that coordinate complex biological processes such as neocortical development.

### miRNA biogenesis

microRNAs (miRNAs) are short 18–22 nucleotide (nt), single-stranded noncoding RNAs that bind primarily to the 3′ untranslated region (UTR) of target mRNAs to repress their translation and stability [19–22]. Biogenesis of miRNA requires sequential steps; miRNA are generally transcribed by RNA polymerase II as immature nuclear precursors called pri-miRNAs, which are stem-loop containing transcripts. In mammals, pri-miRNAs can also be organized in “clusters,” which are transcripts containing multiple stem-loop structures that often give rise to highly similar miRNAs. An example of such a transcript is the miR-17–92 cluster, encoding for some of the best-characterized miRNAs expressed in developing neocortex (see below). Pri-miRNAs are typically processed by a nuclear complex of proteins named the “microprocessor”, which is formed by a type III-like ribonuclease protein, Drosha, and by the RNA binding protein Dgcr8 encoded by the DiGeorge syndrome critical region gene 8. After cleavage, the microprocessor releases 60–100 nt-long hairpin-containing intermediate precursors called pre-miRNAs. Pre-miRNAs are then exported to the cytoplasm by the export 5-Ran GTPase shuttle system. In the cytoplasm, pre-miRNAs are again processed into mature miRNAs by another RNase III-like ribonuclease protein dicer; they are processed into an 18–22 nt duplex. Alternative miRNA biogenesis pathways have also been found in mammals, and currently it has been shown that miRNA biogenesis can occur following non-canonical pathways, which do not require the microprocessor or dicer [23, 24]. After these steps, one strand (or in a few cases both strands) of the dicer-cleaved pre-miRNA is loaded into RNA-induced silencing complex (RISC), which contains RNA binding proteins of the Argonaute family [25].

RISC-loaded miRNA (i.e., mature miRNA) can interact with target mRNAs. This interaction is typically guided by the binding between the 6–8 nt at 5′ end of the miRNA (i.e., the seed region) and the miRNA binding site(s), which can be located in any region of the target mRNA (i.e., 5′, 3′ UTRs or coding region). Recently, seedless miRNA interactions with mRNA targets have also been described [26–28]. Moreover, despite the interaction of a miRNA with its target mRNAs typically inducing the post-transcriptional silencing of the target through inhibition of its translation and/or mRNA destabilization [10], a few miRNAs that can activate translation of targets have also been found [29, 30]. This evidence adds further complexity to the mechanism of miRNA-mediated control of gene expression.

In recent years, it has become apparent that miRNA biogenesis and decay are subject to sophisticated control in the central nervous system (CNS) [23]. For example, it has been shown that brain-derived neurotrophic factor (BDNF), a secreted protein member of the Neurotrophin family of growth factors, can coordinate the translation of specific subsets of mRNAs by potentiating miRNA biogenesis in neurons. Briefly, BDNF can stimulate the maturation of dicer-dependent miRNAs by increasing the levels of dicer protein, and thereby cause a general decrease in the translation of transcripts that are targeted by these miRNAs. In parallel, BDNF also rapidly induces the expression of Lin28 protein, an RNA-binding protein that by preventing the processing of a subset of anti-proliferative pre-miRNA induces pluripotency (see below), and thus causes the selective decrease/loss of mature miRNAs that depend on Lin28. These parallel roles of BDNF are therefore thought to cause a selective upregulation in translation of a certain set of transcripts that are normally repressed by Lin28-dependent miRNAs [31]. On the other hand, it has been shown that synaptic stimulation accelerates miRNA decay in neurons [32]. This evidence therefore suggests that miRNA turnover might play an important role during neocortical development.

### The cell biology of cortical neurogenesis

One of the first steps towards the development the neocortex is the subdivision of the embryonic telencephalon into two halves along the dorso-ventral (DV) axis. The ventral half the subpallium will develop into the basal ganglia, and the dorsal half, also known as pallium, will form the cortex. At the molecular level, the patterning of telencephalon relies on the restricted expression of transcription factors that define the specific regions inside the telencephalon itself, as well as rates of cell proliferation, differentiation and programmed cell death, leading to distinct cell subtypes and morphologies [33]. Initial studies reported only a marginal role of miRNAs in the early patterning of vertebrate forebrain [34–36]. Recent evidence, however, reveals that persistence of domain boundaries between different areas might be tuned by miRNAs. Indeed, a dorsal shift of pallial–subpallial boundary was observed in the developing telencephalon of mice knockouts for two of the three genes encoding miR-9 [37], one of the most abundant miRNAs of the developing cortex. Despite this evidence, the role of miRNAs in the control of the early patterning of the telencephalon still remains a poorly understood aspect of forebrain development, and will require further studies.

Following the initial patterning of telencephalon, cortical development can be roughly subdivided into three main phases, notably, early, mid and late phase (Fig. 1a). These subsequent phases of cortical development are partially overlapping among each other, and some of the subtypes of NSCs/NPCs and cortical neurons that are generated coexist in space and time during neocortical development (Fig. 1a). In the early phase of cortical development, the telencephalonis is composed of a single layer of epithelial-like cells, the neuroepithelial (NE) cells, which are the primary NSCs of the developing cortex and divide at the ventricular (apical) surface of the VZ (Fig. 1a). In this phase, NE cells undergo a massive expansion, mostly by symmetric self-amplifying divisions (i.e., one NE cell generates two daughter NE cells). At the onset of cortical neurogenesis (which in the dorsal telencephalon of the mouse initiates around E10, and about 2 days later in the rat), NE cells progressively start to divide asymmetrically, giving rise to one NE cell plus either a postmitotic neuron or a more differentiated (i.e., fate-restricted) subtype of NPCs that successively replace NE cells (Fig. 1a). In the developing cortex, the earliest postmitotic neurons that are generated by NE cells are mainly the Cajal-Retzius, which migrate out from the VZ toward the basal lamina and accumulate in the preplate (PP, Fig. 1a). The PP is a transient cell structure that is later split into subplate (SP, a developmental zone located immediately below the forming cortical plate, Fig. 1a) and marginal zone (MZ, the most superficial cortical layer that becomes layer I in the postnatal cortex, Fig. 1a) by incoming neurons that form the cortical plate (CP, Fig. 1a). The CP is a cell dense structure of the developing cortex that contains postmigratory neurons and that is expanded in layers II–VI in the postnatal cortex (Fig. 1a). In addition to postmitotic neurons, asymmetrically dividing NE cells also generate fate-restricted subtypes of NPCs (Fig. 1a). The fate-restricted subtypes of NPCs belong to two main categories, those that remain epithelial and divide at the apical surface of the VZ (RG—and short neural precursors, here collectively referred to as apical progenitors); and the progenitors that delaminate from the VZ and divide in the SVZ, or above it [basal progenitors (BP)—also called intermediate, or non-surface, or SVZ progenitors; and outer radial glia (oRG)—also known as intermediate RG, here collectively referred to as BP] [38, 39] (Fig. 1a). The different subtypes of NPCs can be identified by specific morphological characteristics, as well as by expression of a distinct set of proteins [38, 39]. Similarly to NE cells, fate-restricted NPCs can generate progenitors, neurons, or both. However, in contrast to NE cells, fate-restricted NPCs undergo a reduced number of symmetric self-amplifying divisions, and most of them undergo divisions that give rise to differentiated progeny [40] (Fig. 1a). During mid and late phases of cortical development, several waves of different glutamatergic cortical projection neurons are generated by fate-restricted NPCs, either by apical or basal division. Newborn glutamatergic cortical neurons migrate radially from the VZ/SVZ, and by somatic translocation along the basal processes of RG cells, they cross the intermediate zone (IZ), and accumulate in the CP in an inside-out way (Fig. 1a). In particular, the early-born neurons form the deep cortical layers VI–V, while later-born neurons accumulate above the deep layers, forming the upper cortical layers IV–II [6, 41] (Fig. 1a). Finally, later aspects of cortical development include, but are not limited to, neurite outgrowth and the dendritic elaboration of cortical neurons, which lead to the formation of short and long projections, and synaptogenesis with their target sites. In rodents, these aspects are mostly concluded in the first 2–3 postnatal weeks [42] (Fig. 1a). In addition to cortical neurons, several types of glial cells, such as astrocytes and oligodendrocytes, are present in the adult mammalian cortex. In rodents, despite recent evidence indicating that astrocytes precursor cells are generated during embryonic corticogenesis [43], most of the astrocyte and oligodendrocyte cells can be detected after the disappearance of cortical NPCs, in the early postnatal cortex [5]. Given that the role of miRNAs in these later aspects of corticogenesis has been extensively reviewed elsewhere [13–17], here we will not discuss these aspects in detail.

In short, neocortical development is a complex process that results from the execution of a precise developmental program that is controlled in a spatially and temporally specific manner. Extracellular signals and networks of intrinsic/intracellular factors that govern this developmental program require a precise and coordinated orchestration of their expression, and miRNAs are certainly part of this mechanism.

### Dynamic expression of miRNAs during neocortical development

It is now clear that a tightly regulated event, such as neocortical development, needs a coordinated expression of genes and miRNAs that converge to maintain the proper developmental program. Several studies have profiled miRNA expression in embryonic neocortex of rodents, and provided a crucial first step toward the identification of the possible function(s) of miRNAs in the control of cortical development [44–48]. These studies identified a cohort of miRNAs whose expression dynamically changes during cortical development (Fig. 1b), and provided evidence that in developing cortex some miRNAs share similar dynamics of expression (Fig. 1b), suggesting that co-expressed miRNAs might act cooperatively to modulate the activities of signaling networks that are required during specific phases of cortical development (Fig. 1a). According to these data, the expression pattern of miRNAs in developing cortex can be roughly classified into four main trends (Fig. 1b): (1) miRNAs that are continuously expressed throughout development (e.g., some miRNA members of the let-7 family and miR-9/9*); (2) miRNAs that start to be expressed during early development and decline their expression during development, or around perinatal stage (e.g., miR-125b, miR-181 family, and miRNAs encoded by the *miR*-*17*-*92* cluster, and by its paralogous genes *miR*-*106a*-*363*, *miR*-*106b*-*25*, collectively referred to as miR-17-92 subfamily); (3) miRNAs that start to be expressed in mid or late development and then remain expressed also in postnatal brain (e.g., miR-124, miR-128, etc. ); and (4) miRNAs that are specifically expressed during a short period during development. The latter category is particularly interesting, because it might control specific stages of cortical development such as cell proliferation, viability, neuronal differentiation, migration, and neuronal network formation.

### MiRNAs that are continuously expressed throughout corticogenesis

The let-7 family of miRNAs is among the miRNAs that show a continuous expression throughout cortical development (Fig. 1b). let-7 is encoded by the *Lethal*-*7* (*let*-*7*) gene, one of the founding miRNAs discovered in the nematode worm *Caenorhabditis elegans* (*C.* *elegans*), and is evolutionarily conserved in vertebrates [49]. In mammals, the let-7 family of miRNAs comprises several mature miRNA sequences that differ only in a few nucleotides. The small differences in their seed regions are thought to discriminate let-7 family members for their specific target genes, thus leading to distinctive biological consequences [50]. let-7 family members play significant roles in proliferation of NSCs/NPCs and in the control of neurogenesis [50] (see below). Mature miR-9 and -9* also show high expression levels throughout cortical development (Fig. 1b). Interestingly, miR-9 and -9*share the same pre-miRNA precursor transcript. As introduced above, typically only one mature miRNA can be detected as a stable product cleaved from a double strand pre-miRNA. However, in mammals the precursor for these miRNAs is transcribed by three loci (i.e., miR-9-1, miR-9-2 and miR-9-3), which are all co-expressed in the developing mouse cortex [37]. Therefore, it is possible that miR-9 and -9* derive from the cleavage of independent pre-miRNA precursors during brain development. Despite this hypothesis, the exact mechanism of miR-9 and -9* biogenesis in developing cortex remains obscure.

### Some sets of miRNAs share dynamic expression patterns during corticogenesis

As introduced above, the early phase of cortical development is characterized by the fast proliferation and expansion of NSCs/NPCs. In the rat cortex, this phase occurs roughly between embryonic day 10 (E10) and E11 (Fig. 1a). In recent study, Yao et al. [48] performed miRNA profiling in developing and postnatal rat cortical tissues [from E10 to post-natal day 28 (P28)], and found that nearly 40 % of all miRNAs expressed during cortical development had the highest abundance at E10, while decreasing at later stages (Fig. 1b). Among those miRNAs, miR-34c, -152, -219-5p, -301b, -449a, -451 and -532-5p were tenfold more abundant at E10 than at any other stage, providing a hint that those miRNAs may play important roles in the regulation of NSC/NPC proliferation and viability [48]. In another study from Nielsen et al. [46], it has been reported that only 7. 2 % of miRNAs underwent significant changes between E11 and E13, whereas the majority of them did not change expression during this developmental time window. It should be noted, however, that some of the miRNAs found to be highly expressed between E10 and E11 in Yao’s study, such as miR-20b*, -126, -143, -183, -199a, -200b/c, -214, -222 and -292-3p, are not overlapping with those of Nielsen’s study [46, 48]. This discrepancy might be due to the different sensitivity of the methods used, to the different cell sources, or to the existence of strain-specific miRNAs. Further detailed discussion about the systematic comparison of commercially available miRNA profiling platforms, as well as RNA extraction and quality control methods, can be found in a recent paper from Git and colleagues [51].

Around E12–E13 (i.e., at the onset of cortical neurogenesis in rats), several changes in the expression of miRNA are observed (Fig. 1b) [48]. At this stage, some miRNAs that are enriched in the early phase of cortical development (e.g., the miR-181 family and miR-199a) gradually start to decrease, and eventually their expression stops before birth, suggesting a possible role of these miRNAs in control of proliferation and possibly other aspects of early cortical development (Fig. 1a) [48]. At the same stage, other miRNAs are at the peak of their expression (e.g., miR-7, -7a and miR-191, Fig. 1b), suggesting a possible role for these miRNAs at the onset of neurogenesis (Fig. 1a) [48]. Conversely other miRNAs, such as those encoded by the *miR*-*17*-*92* subfamilies of miRNAs (not shown), and others belonging to a well-characterized category of CNS-enriched miRNAs, namely miR-99a -124a and miR-266, start to be upregulated (Fig. 1b) [46]. In addition to these miRNAs, between E13 and birth, other miRNAs start being expressed (Fig. 1b), followed by their downregulation after birth [44]. The miRNAs encoded by the *miR*-*17*-*92* cluster are amongst the best-characterized miRNAs in mammals, and are expressed in many tissues. At the functional level, knockout mice for the *miR*-*17*-*92* cluster (and its paralogous genes) display early embryonic lethality, and miRNAs encoded by this cluster play essential roles in the control of NSC/NPC self-renewal, and subtype specification [52].

In the developing rat cortex, between E21 and birth most of the glutamatergic cortical neurons reach their final laminar destination and start projecting axons and dendrites toward their final targets (Fig. 1a). At this stage, the expression of miR-19b decreases rapidly; miR-137 shows a peak in its expression; whereas other miRNAs such as let-7b, miR-128 and miR-185 start to be expressed (Fig. 1b) and their expression increases in the postnatal days (especially between P14 and P28) [48]. In rodents, major sensory inputs are established and most glial cells are generated during the first 2–3 postnatal weeks [5]. For example, eye opening occurs around P13 and is thought to result in activity-dependent neuronal remodeling. Consistently, some of the miRNAs that are enriched during late embryogenesis, or at the perinatal stage, such as miR-128 (Fig. 1b) and miR-29a (not shown), tend to increase over time [48], suggesting a role for these miRNAs in late aspects of brain development, such as fine control of cortical connectivity, or gliogenesis.

### Detection of miRNA expression in corticogenesis: what’s next?

miRNA profiling is very informative and has allowed us to gain important information about temporal expression of miRNAs during corticogenesis. However, this approach also has limitations. For example, conventional miRNA profiling does not provide information with respect to spatial expression of miRNAs. Moreover, the high heterogeneity of the cells types present in the developing neocortex, or during the early postnatal weeks (when major sensory inputs are established and most glial cells are generated [5]), might contribute to the changes observed in miRNA profiles. In order to have a complete picture of the pattern(s) of miRNA expression in the various cortical cell types, some recent studies have started to perform cell-type-based analysis of miRNA profiles in the mouse brain [53, 54]. In particular, these studies profiled the expression of miRNAs in various neuronal subtypes, such as glutamatergic and GABAergic neurons [53], or in neurons and glia [54]. It would also be interesting to apply this approach to perform profiling of miRNA expression in specific subpopulations of NSCs/NPCs in developing cortex. For example, given that oRG has been implicated in the evolutionary expansion of the neocortex in primates and human, in particular in the generation of the outer SVZ (a transient germinative layer that is typical of the development of gyrencephalic cortex [38, 39]), future comparative studies on miRNA expressed in oRG might shed new light on the molecular mechanisms at the base of expansion and evolution of the neocortex.

On the other hand, techniques such as negative sensors for miRNAs [55, 56], in situ hybridization [57, 58], or the use of transgenic organisms expressing green fluorescent protein (GFP) under the control of promoters of miRNAs genes [59] allow us to gain insight into spatial and temporal dynamics of the expression of a given miRNA. By using these techniques, it was recently uncovered that the expression pattern of miR-124, one of the most abundant miRNAs of the mammalian brain and generally regarded as being specifically restricted to post-mitotic neurons in the developing cortex [60, 61], starts its expression in NPCs [56, 62, 63].

Thereby, the use of cell-type based miRNA profiling in combination with techniques providing spatial information and dynamics of miRNA expression will be important to gain a better insight into possible functions of miRNAs in the control of specific aspects of cortical development.

### miRNA depletion approach to investigate the global requirement for miRNAs during neocortical development

The correlation of some sets of miRNAs with distinct phases of cortical development (Fig. 1) suggests that co-expressed miRNAs might coordinate gene expression to regulate specific aspects of cortical development, such as NSC/NPC expansion and their differentiation.

How is it possible to functionally investigate the role of co-expressed miRNAs during cortical development in vivo? For this purpose, several studies have used depletion of miRNAs, by means of genetic inactivation of the RNase III enzyme dicer or other essential proteins for miRNA biogenesis such as Dgcr8.

Mice null for dicer, or Dgcr8, are not viable and die in utero before the onset of cortical neurogenesis, indicating that miRNAs are critical for mammalian development [64, 65]. To bypass the early embryonic lethality and investigate the global role of miRNAs in specific phases of cortical development (or cortical cell types), several mouse mutant lines carrying conditional deletions of dicer have been created. In these studies, conditional deletion of Dicer was obtained in vivo with Cre-recombinase driven by *Foxg1*, *Emx1*; *Nes*, *Nex*, human *GFAP*, and *Camk2* promoters [36, 66–78].

Overall, conditional deletion of dicer in the embryonic neocortex resulted in gross anatomical abnormalities, and provided evidence that miRNAs regulate important aspects of cortical development, such as cell viability, self-renewal and commitment (i.e., the program engaged by stem or progenitor cells that leads to their differentiation) of NPCs, as well as differentiation, migration and maturation of glutamatergic cortical neurons, and in turn, the proper formation of cortical layers.

### Cell loss upon conditional deletion of dicer in embryonic neocortex

In embryonic neocortex, conditional deletion of dicer in vivo (although with some noticeable differences depending on the onset of Cre-recombinase expression) generally causes loss of neurons, and in some cases also loss of NSC/NPC pools [36, 66–78]. Overall, a plausible explanation for cell loss upon conditional deletion of dicer in embryonic neocortex is increased cell death. However, which specific cell types are actually lost, as well as the mechanisms responsible for cell death upon dicer deletion, are still unresolved questions (see limits of the global miRNA-depletion approach, below).

Cell loss could be triggered, for example, by impaired differentiation of dicer-deleted NSCs/NPCs (and newborn neurons), which might fail to exit the cell cycle upon differentiation and therefore undergo cell death. In some studies, the loss of neurons was due to increased apoptosis [36, 66–72, 75]. However, in other studies and in agreement with in vitro evidence [79], neuronal loss observed upon dicer deletion in vivo is not always due to a dramatic increase in apoptosis [67, 73, 76, 78, 80, 81].

On the other hand, cell loss in dicer-deleted embryonic cortices might also be due to the progressive decline of proliferation of dicer-deleted NSCs/NPCs, leading to their proliferative arrest and death. Remarkably, despite self-renewal of NSCs/NPCs seeming to be less affected by dicer deletion than cell fate transitions [68, 79, 82] (see also cell fate transitions and developmental maturation of NSCs/NPCs below), several studies reported progressive arrest in NSC/NPC proliferation upon dicer deletion in embryonic cortices [36, 66, 68, 69, 71, 73, 75]. Two recent studies characterized in detail the proliferation of dicer-deleted NPCs during late cortical and hippocampal development, and both studies found that miRNAs are essential for maintenance of NPC pools [71, 73]. In the first study, reduced proliferation of hippocampal NPCs was observed at E15. 5, upon dicer deletion obtained with *Emx1* and *Nestin* promoters [71]. In the second study, dicer deletion was obtained with the human *GFAP* promoter, and this resulted in a significant impairment in RG cell proliferation at P15 and P40. This proliferation defect was mostly attributed to “High-temperature requirement A serine peptidase 1” (HtrA1) gene product, whose overexpression in the developing neocortex recapitulated some of the phenotypes observed in dicer deleted cortices. The results of these two studies indicate that at late developmental stages, miRNAs functions are essential for the maintenance of the NPC pool in both hippocampus and cortex.

It remains to be seen whether the increased apoptosis observed at certain stages of development, or the limited sensitivity of self-amplifying NSCs/NPCs to the dicer deletion, reflect a real characteristic of the miRNA pathway (e.g., that might be required/dispensable for maintenance of the NPC pool at certain developmental stages), or is due to some technical limitation of the conditional dicer deletion approach (see limits of the global miRNA-depletion approach, below).

### Cell fate transitions and developmental maturation of NSCs/NPCs are impaired upon conditional deletion of dicer in embryonic neocortex

Perhaps the most recurrent theme resulting upon selective deletion of dicer in cortical NSCs/NPCs is that miRNA depletion often impairs cell fate transitions (e.g., the transition from apical to BP, or the differentiation of NSCs/NPCs to cortical projection neurons) or developmental maturation toward more restricted progenitor cell types (e.g., progressive restriction of RG competence). In agreement with this statement, a wide spectrum of defects, such as premature, delayed, or reiterated generation of certain subtypes of neural stem and progenitors cells (both apical or BP), neurons, or glia, has been reported upon dicer deletion in developing neocortex [36, 66, 68, 69, 71, 73, 75, 76, 81]. This evidence strongly supports our initial observation that in developing mouse, neocortex miRNA functions are less required for self-amplification of NSCs/NPCs, but are particularly important during cell-fate transitions [68]. In particular, miRNA-depleted embryonic NSCs from dicer-deleted cortices can self-renew for several days in vivo [68] or in vitro [79, 82], but are unable to differentiate in neurons or astrocytes, and exhibited a marked dependency on exogenous mitogens for survival [68, 79, 82]. These data are also consistent with similar observations during the differentiation of dicer-deleted murine embryonic stem cells [65, 83, 84] in vitro.

More recently, it has been observed that conditional deletion of dicer also impairs the developmental maturation of NSCs/NPCs. In particular, it has been shown that dicer deletion results in the progressive restriction of RG competence [76]. Another example is the transition between the pre-neurogenic NE cells and the neurogenic RG [36]. Given that there are very few known mechanisms implicated in the regulation of either of these processes, it is therefore particularly interesting that miRNAs might also be involved in these important aspects of cortical development.

In short, these common themes for miRNA function in developing cortex are reminiscent of those observed for the founding miRNAs lin-4 and let-7 in C. *elegans*; that clearing and repressing previously expressed transcripts facilitates progression to the following developmental stage [19–22].

### Defective cortical lamination and migration upon dicer deletion in embryonic neocortex

As introduced above, the cerebral cortex is a highly organized laminar structure that results from the precise positioning of subsequent waves of cortical glutamatergic neurons that are generated in the course of cortical development (Fig. 1a). The first study addressing this aspect in detail reported laminar defects in the postnatal neocortex upon *Emx1*-Cre mediated dicer deletion [68], suggesting that radial migration of cortical glutamatergic neurons was impaired upon miRNA depletion. Consistent with this finding, misplacement of neurons in dicer-deleted embryonic or postnatal neocortex was reported in a number of studies [36, 69, 71, 75, 76]. Several mechanisms might explain the various defects in neuronal migration that have been reported in dicer-deleted cortex. For example, loss of radial RG cells, which has been observed upon conditional dicer deletion, might affect the integrity of the scaffold used during somatic translocation of radially migrating cortical projection neurons (Fig. 1a), thus causing their retention in the deep cortex [36, 68]. Another example is the loss of Cajal-Retzius cells, which have been shown to impair radial migration of cortical neurons [85]. Indeed, lost or misplaced Cajal-Retzius cells in the developing cortex have been observed upon conditional deletion of dicer [71, 75]. Finally, defective coordination of leading process extension and branching in neurons has also been observed upon dicer deletion [78, 86]. Given the intricacy of the signaling and pathways involved in control of the radial migration of neurons in developing cortex, it is very difficult to gain insight into the role of miRNAs in the regulation of radial migration of cortical projection neurons by using conditional deletion of dicer. This aspect therefore still remains poorly understood.

### Conditional deletion of Dgcr8 in embryonic neocortex

In addition to dicer deletion, depletion of miRNAs can also be obtained by conditional deletion of the Dgcr8 gene. A recent study compared the phenotypes in the postnatal brain of mice in which the conditional deletion of dicer and Dgcr8 in post-mitotic neurons of the embryonic and postnatal cortex was mediated by Cre-recombinase driven by *Camk2* promoter [72]. The loss of Dgcr8 resulted in a later lethality, milder structural abnormalities, and less apoptosis relative to that from dicer loss. Deep sequencing of small RNAs from postnatal hippocampus and cortex isolated from the conditional Dgcr8 deleted mice identified multiple non-canonical miRNAs, including miRtrons that were differentially expressed relative to dicer-deleted animals, suggesting a diverse population of highly expressed non-canonical miRNAs that together are likely to play important functional roles in post-mitotic neurons. It would be interesting to see whether the conditional deletion of Dgcr8 in NSCs/NPCs during corticogenesis will also result in a milder phenotype compared to conditional deletion of dicer.

Recently, Dgcr8 gene has also gained momentum in the physiopathology of neural defects observed in the DiGeorge syndrome, a genetic disorder that in humans originates from the micro deletion of the region q11.2 of chromosome 22 encoding for about 30 genes, including the DGCR8 gene [87–89]. These studies further highlight the importance of miRNAs and miRNA biogenesis pathway genes in complex neural diseases.

### Limitations of the global miRNA-depletion approach

Taken together, the in vivo studies of miRNA function, by conditional deletion of essential genes for miRNA biogenesis, revealed that miRNAs are crucial regulators of cortical development, and also underscored that miRNAs are part of a potent (and still underestimated) mechanism that can greatly impact on behavior and may predispose to brain disorders. On the other hand, this approach has some inconveniences, such as the subtle differences in the phenotypes observed in embryonic cortices of dicer-deleted mice that might be due to the promoter used to induce Cre-expression (see for example [68, 69]), or by the fact that dicer ablation might be incomplete/delayed in certain cell types, or developmental times. Moreover, complete depletion of some miRNAs might require a long time upon dicer deletion [79]. Furthermore, one should also consider that additional functions of the microprocessor might contribute to the observed phenotypes. In agreement with the latter possibility, a recent study reported that knockdown of genes encoding for components of the microprocessor complex in NPCs of the developing mouse neocortex resulted in a loss of stem cell character and in their precocious differentiation, whereas knockdown of dicer did not, suggesting that the microprocessor regulates neurogenesis in a miRNA-independent way [90]. Finally, given the overwhelming amount of apoptosis observed upon dicer ablation in some studies, and that clearance of the apoptotic cells in the developing tissues is typically very rapid, it is sometimes very difficult to draw conclusions. Despite these limitations, which might complicate the analysis and interpretation of results, the approach of conditional deletion of essential moieties for miRNA biogenesis still remains widely used to investigate the functional role of miRNAs in mouse CNS, and indeed, several studies have identified specific miRNAs and targets responsible for some of the dicer phenotypes [66, 70, 73, 74, 91–93].

### Convergent miRNA actions on targets coordinate gene networks in the developing cortex

Proper cortical development requires the generation of the appropriate numbers and types of cells from common progenitor pools, and the right positioning of neurons in the cortical plate. This is achieved by integrating multiple intrinsic and extrinsic signals governing the relationships of self-renewal, commitment, survival, differentiation, delamination/migration, attractions/repulsions, etc.

**Fig. 2:**
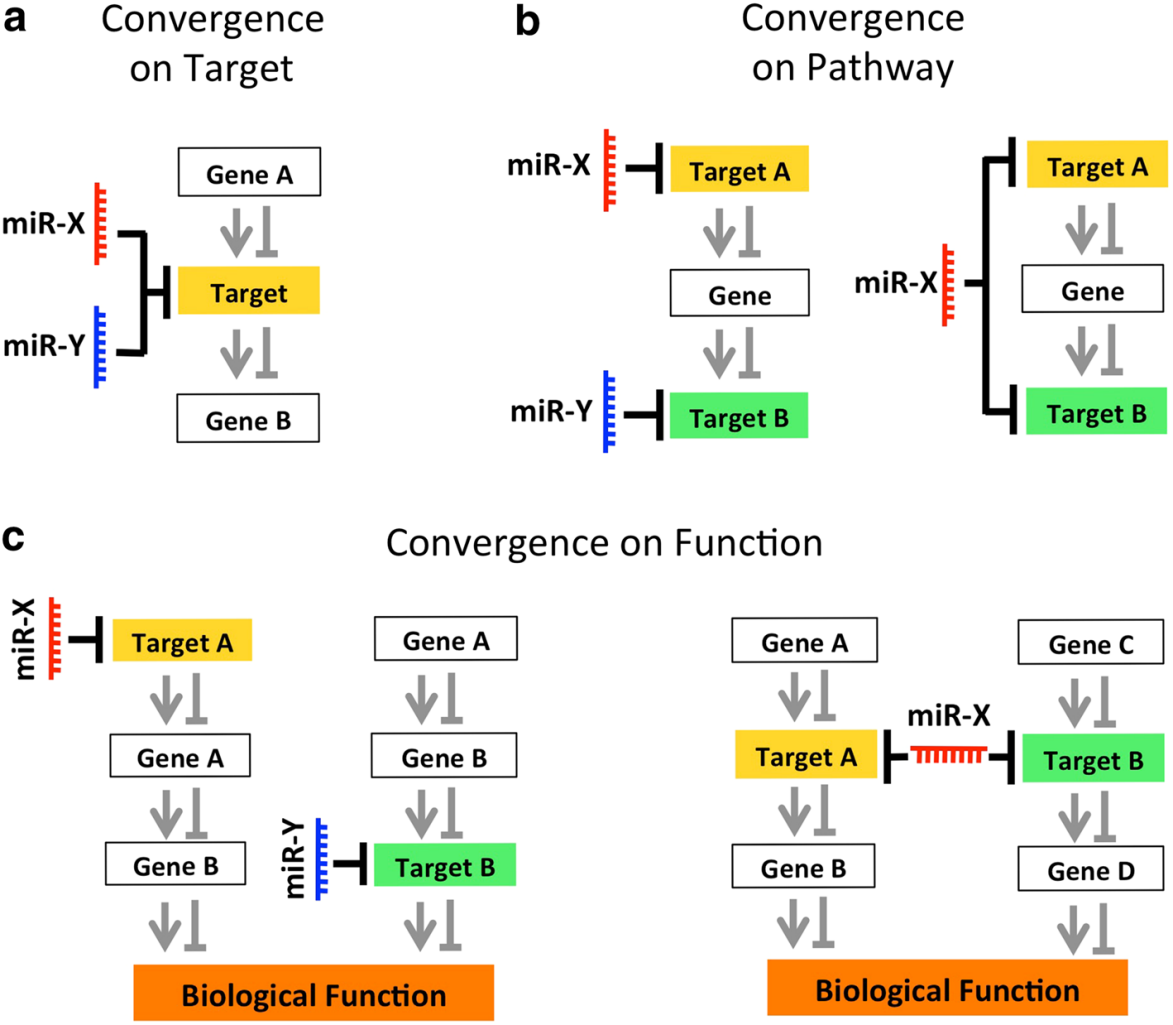
Examples of convergent miRNA actions on targets. miRNAs might act through convergent actions to orchestrate intrinsic and extrinsic signals during cortical development. Examples of convergent miRNA actions include, but are not limited to, the simultaneous co-regulation of a single target by one or more co-expressed miRNAs [convergence on target, previously defined by Schouten and colleagues as miRNA cooperativity (**a**)]; coordinated regulation of different target encoding for proteins that are acting in the same pathway [convergence on pathway, (**b**)]; or coordinated regulation of target encoding for proteins that are exerting redundant functions [convergence on function, (**c**)]. Convergent miRNA actions might compensate for the mild degree of miRNA-dependent regulation of mRNA targets, and contribute to achieving the precise execution of the molecular program that leads to proper cortical development

Accumulating evidence shows that a single miRNA can act in parallel on several targets either in one cell, in different cells, or at different developmental time points, thus coordinating the intrinsic and extrinsic signals that control cortical development. On the other hand, by using a proteomic approach, it was demonstrated that the extent of repression mediated by a single miRNA is surprisingly mild [94, 95]. With such a mild regulation, it is therefore unclear how a miRNA can provoke a meaningful functional change in a biological process. An attractive possibility to solve this apparent paradox is that cooperation between co-expressed miRNAs might compensate the fine-tuned mRNA regulation mediated by a single miRNA, thus exerting a broader impact on gene expression compared to a single miRNA (Fig. 2). This scenario is supported by experimental evidence indicating that different miRNA binding sites in the same 3′UTR can potentiate the degree of translational repression of a single miRNA [96–100]. In a recent review from Schouten and colleagues [18], the positive interaction of two or more individual miRNAs, or one individual miRNA, acting on multiple seed regions on the same 3′UTR was defined as “miRNA cooperativity,” a concept that highly resembles the “cooperative binding” of ligands to a single receptor protein, or of transcription factors to a single promoter in DNA.

Although we agree with the strict definition of miRNA cooperativity [18], it does not account for the abundant evidence of individual miRNAs co-regulating different target genes in parallel (e.g., see Lim et al. [101] and Conaco et al. [102]), and that some of the co-regulated genes might exert redundant functions (see below).

We therefore propose the wider concept of “convergent miRNA action,” which we define as “the synergic action of one or more individual miRNAs that by acting on different seed regions in one or more target genes results in a regulatory effect”. In our definition, the synergic action of miRNAs might affect a single gene, molecular pathway, and in turn different biological functions. Examples of convergent miRNA actions include, but are not limited to, the simultaneous co-regulation of a single target mRNA by two or more miRNAs (Fig. 2a, previously defined by Schouten and colleagues as “miRNA cooperativity” [18] and here referred to as convergence on target, for internal consistency); coordinated regulation of different targets encoding for proteins that are acting on the same pathway (Fig. 2b, referred to as convergence on pathway); or coordinated regulation of targets encoding for proteins that are exerting similar functions (Fig. 2c, referred to as convergence on function).

In order to be effective, convergent miRNA actions must occur not only between miRNAs with shared seed sequences (e.g., members of the same family of miRNAs, such as for example let-7 family), but also amongst miRNAs with no shared seed identity. Moreover, convergent miRNA actions might involve the capability of the co-regulated genes to feedback on these miRNAs to regulate their expression and function. For example, convergent miRNA actions might result in concerted actions of multiple components of the miRNA turnover pathway, as well as of the effector proteins that mediate miRNA functions. However, our current lack of understanding of the mechanisms that regulate changes in miRNA abundance and functions during corticogenesis does not allow us to describe these aspects in detail. Finally, in the developing cortex, miRNA functions are particularly required during cell-fate transitions and developmental maturation [36, 66, 68, 69, 71, 73, 75, 76, 81], and in NSCs/NPCs, several miRNAs have been found to control the balance between self-renewal, developmental maturation to more restricted progenitor subtypes and commitment toward neuronal fate, as well as their survival. These aspects are interdependent during corticogenesis, and might be orchestrated by convergent miRNA actions on multiple genes and pathways that control proliferation, differentiation and survival.

**Fig. 3:**
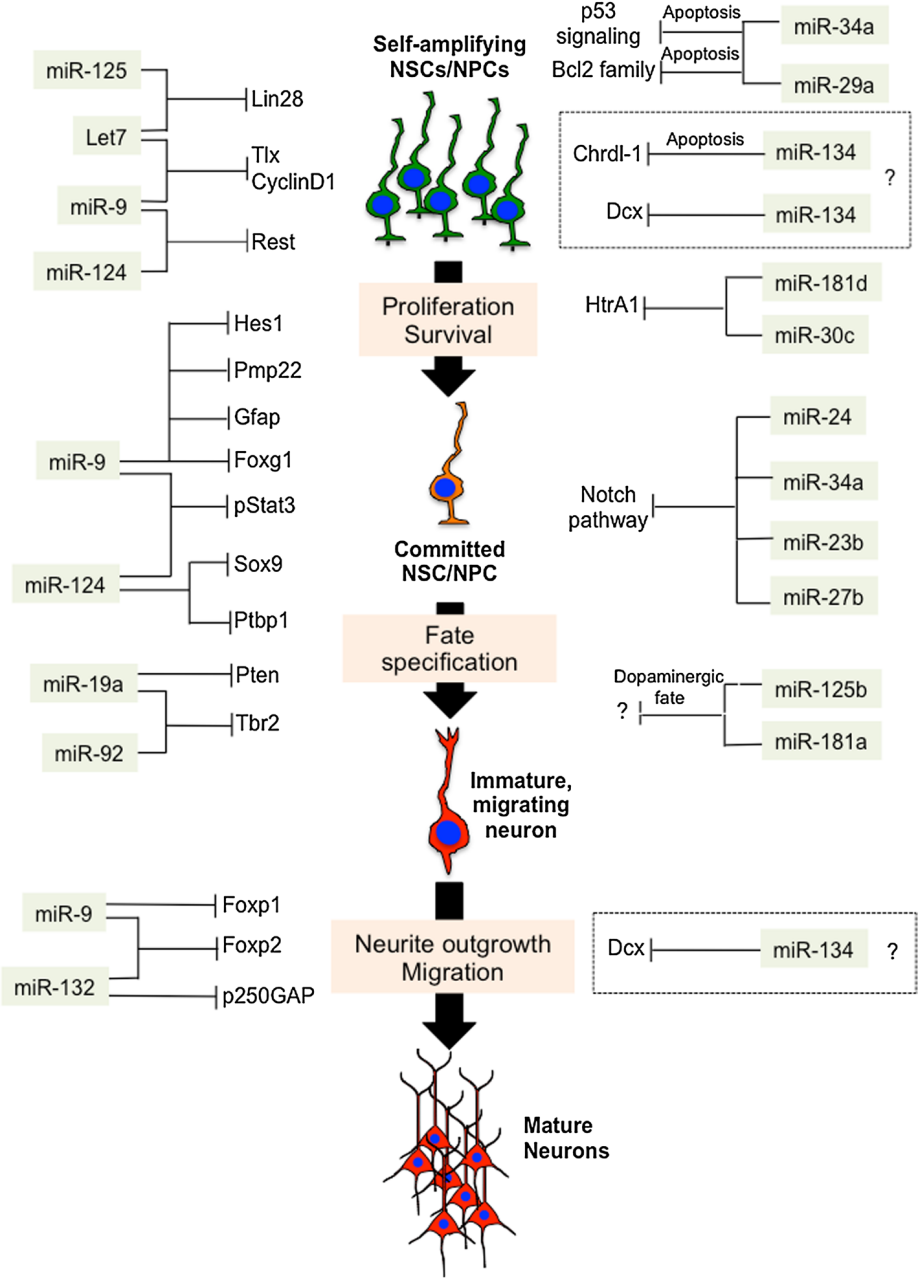
Convergent miRNA actions coordinate gene networks in the developing cortex. Convergent miRNA actions direct the expression of gene networks and modulate important aspects of cortical development, such as proliferation/survival, commitment and fate specification in neural stem and progenitor cells (NSCs/NPCs), neurite outgrowth, and migration in immature cortical glutamatergic neurons. The identification of convergent miRNA actions is potentially one of the keys to disclosing how miRNAs achieve the precise orchestration of complex biological processes such as neocortical development, and is consistent with the proposed “tuning” function of animal miRNAs. *Dashed boxes* indicate convergent miRNA actions that are not sufficiently supported by present data

In the remaining part of this review, we will describe known and possible examples of convergent miRNA actions that coordinate the generation and migration of pyramidal-projection glutamatergic neurons, and the initial formation of cortical layers in the embryonic neocortex of rodents (Fig. 3). This is still a poorly understood layer of complexity in miRNA signaling, but potentially one of the keys to disclosing how miRNAs achieve the precise execution of the molecular program that lead to proper cortical development.

### Convergent miRNA actions control proliferation and commitment of NSCs/NPCs

The first example of convergent miRNA action involves the founding miRNAs lin-4 (miR-125 in mammals) and let-7 (let-7 family in mammals) (Fig. 3) [19–22]. Sequences and functions of miR-125 and let-7 are highly conserved throughout the animal phyla [103, 104]. miR-125 and let-7 converge on the common target lin-28, a potent induced or pluripotency [105], regulating proliferation and commitment of NSCs/NPCs. In particular, miR-125, let-7 and their common target lin-28 are part of an auto-regulatory circuit. In this circuit, Lin-28 protein binds to the precursors of several miRNAs (including some members of the let-7 family of miRNAs), inhibiting their processing by dicer, and by this means promoting self-renewal of NSCs/NPCs [106]. On the other hand, miR-125 and let-7 converge on lin-28 mRNA and by this means reduce Lin-28 protein levels, thus releasing the inhibition of let-7 maturation and promoting the commitment of NSCs/NPCs toward neuronal fate [107].

let-7 and miR-9 control proliferation and commitment of NSCs/NPCs by converging on a common target, and by converging on different target genes with redundant function (Fig. 3). Briefly, let-7 and miR-9 converge on the common target TLX, an essential regulator of NSCs/NPCs proliferation and commitment. The convergent action of let-7 and miR-9 reduces levels of TLX protein, and thereby inhibits self-renewal of NSCs/NPCs, accelerating their differentiation toward neuronal fate [108–110]. TLX also feeds back on miR-9 by repressing the transcription of the miR-9 precursor, and by this means limits the expression of this miRNA in proliferating NSCs/NPCs. On the other hand, let-7b (a member of let-7 family of miRNAs) represses the cell cycle regulator CyclinD1, and miR-9 represses the basic helix-loop-helix transcription factor Hes1, respectively (Fig. 3) [110–112]. Given that both genes are positive regulators of NSC self-renewal, the parallel repressive actions of let-7b and miR-9can be regarded as exemplary of convergence on function that modulates proliferation and stimulates commitment of NSCs.

Several additional examples of convergent miRNA actions involve members of the let-7 family of miRNAs. These convergent actions of let-7 on genes with redundant functions, such as RAS, high mobility group AT-hook 2 (HMGA2) and CDC25 (not shown), have been shown to attenuate proliferation [113–115]. Recently, the let-7/Lin-28 pathway has also been linked to the pluripotency factor SOX2, thus proving further complexity to the role of this miRNA in keeping in balance of self-renewal and commitment in NSCs/NPCs (not shown) [116]. Another example of convergence on target to control NSC/NPC self-renewal in developing cortex involves miR-181d and miR-30c and their common target HtrA1 (Fig. 3) [73]. In this study, it was found that miR-30e and miR-181d converge on the 3′UTR of HtrA1 transcript, repressing its translation, and in vivo overexpression of these two miRNAs rescued RG proliferation defects in dicer deleted cortices, thus suggesting that this mechanism plays a major role in the maintenance of RG cells pool [73].

### Convergent miRNA actions control commitment toward neuronal fate by repressing non-neuronal fates in NSCs/NPCs

Convergent miRNA actions in the embryonic neocortex have also been shown to control the commitment of NSCs/NPCs toward neuronal fate by blocking the expression of non-neuronal genes. The convergent actions of miR-9 and miR-124 are exemplary of multiple combinations of convergence on target (Fig. 2a), convergence on pathway (Fig. 2b) and convergence on function (Fig. 2c). In particular, miR-9 and miR-124 can induce commitment of NSCs/NPCs toward neuronal fate by converging on the common target RE1-Silencing Transcription factor (REST), co-repressing its expression (Fig. 3) [102, 117–119]. REST is a master repressor of neuronal programs in non-neuronal tissues [also known as neuron-restrictive silencer factor (NRSF)] [120–124]. Moreover, miR-124 alone can exert a pro-neurogenic function by limiting the expression of SCP1, a phosphatase that is a component of the REST/SCP1 pathway that participates in the repression of neuronal genes in non-neuronal tissues [117], and by preventing the expression of the polypyrimidine tract-binding protein 1 (PTBP1, a repressor of neuron-specific alternative splicing [125, 66] (Fig. 3). The important role of miR-9 and miR-124 in inducing neuronal fate is further confirmed by recent studies in which overexpression of these miRNAs has been found to facilitate the direct conversion of human fibroblasts into neurons by modifying the subunit composition of the BAF chromatin-remodeling complex, and by repressing PTB [126, 127].

In addition, miR-9 and miR-124 can induce neuronal fate by preventing gliogenesis. Indeed, an important step for the acquisition of the glial cell-fate is the phosphorylation of the signal transducer and activator of transcription 3 (Stat3) (Fig. 3). By overexpressing miR-9 and miR-124, the levels of phosphorylated Stat3 decrease, so the rate of glial cell-fate determination is diminished [128]. At the same time, miR-124 can also repress Sox9, a transcription factor that directs differentiation of NSC/NPCs toward gliogenic fate [129]. Finally, miR-9 is highly expressed in mouse oligodendrocyte precursor cells, and in these cells it targets the 3′UTR of the mRNA encoding peripheral myelin protein Pmp22, repressing its expression (Fig. 3). Thus, together with the inhibition of the expression of the astrocyte-specific protein GFAP, miR-9 may repress expression of non-oligodendrocyte lineage related proteins in oligodendrocytes, and therefore function as a guardian to maintain oligodendroglial cell identity [128]. In short, this evidence, together with the capability of miR-124 to downregulate the expression of large numbers of targets [101, 102], certainly places miR-124 and miR-9 at the center of multiple convergent pathways that are important for the control of neocortical development.

Additional studies have provided evidence that miRNAs can also balance self-renewal and commitment in developing cortex by converging on the Notch pathway. Notch1, Numb, Numbl are critical players in the complex regulation of cortical neurogenesis, balancing progenitor self-renewal, proliferation and fate specification in NSCs/NPCs [130]. Initial evidence, obtained in in vitro differentiating neuroblastoma cells SH-SY5Y indicated that miR-34a and miR23b/24/27b, which are upregulated in these cells upon neuronal induction, converge and repress Notch1 (Fig. 3) [131]. More recently, conditional deletion of dicer induced by h*GFAP*-Cre in Bergmann glia (BG) in developing cerebellum, resulted in a smaller and less developed cerebellum, accompanied by aberrant BG morphology, in vivo. These effects were attributed to miR-9 mediated repression of brain lipid binding protein (Blbp), a well-known downstream target gene of Notch1 protein [132]. In another recent study, the over-expression of miR-34a in NPCs significantly reduced the neuron yield upon induction of differentiation in vitro [133]. Interestingly, several of the potential targets of miR-34a that were found in this study belong to the Notch pathway (Fig. 3). In particular, in self-amplifying NPCs, miR-34a repressed the mRNA and protein levels of Numbl (a negative regulator of the Notch signaling [130]), and of two downstream pro-neural genes, NeuroD1 and Mash1 (usually blocked by Notch signaling), whereas Notch1 and Cbf1 transcripts were enhanced by miR-34a overexpression [133], suggesting that miR-34-mediated control of NPCs self-renewal and commitment might be exerted through convergence on multiple genes of the Notch pathway (Fig. 3) [133]. In short, the Notch pathway appears to be a critical hub for miRNA-mediated control of the balance between self-renewal and commitment toward neuronal fate in developing mammalian cortex.

### Convergent miRNA actions balance survival and differentiation

Regarding the miRNA-dependent control of cortical progenitor survival during development, although there is some evidence suggesting that miRNAs can directly regulate pro-apoptotic and anti-apoptotic genes, other evidence indicates that these miRNAs also regulate additional aspects, such as cell proliferation, cell cycle exit and commitment toward neuronal fate. Convergent miRNA actions between members of the miR-34 family and miR-29a suggest possible examples of convergence on pathway (Fig. 2b) and convergence on function (Fig. 2c). miR-34a and miR-29a converge on the p53 pathway to establish the balance between cell proliferation and cell death (Fig. 3). However, both miRNAs are also involved in the control of differentiation of neuronal cells (Fig. 3). This evidence suggests that miR-34a and miR-29a in NSCs/NPCs might be part of a checkpoint mechanism that activates cell death program in response to aberrant differentiation. In particular, miR-34a is a highly conserved pro-apoptotic miRNA that inhibits cell proliferation in normal cells, and is induced by p53 in response to DNA damage. MiR-29a has also been implicated in apoptosis by targeting members of the Bax family of anti-apoptotic proteins and p85α, which results in activation of p53 (Fig. 3), and miR-29a is downregulated in neurons undergoing apoptosis [134–143]. Finally, miR-34 has been shown to target Sirt1 and also to be regulated by p53, which in turn is activated by Sirt1 [144–146]. These data, together with the increased cell death observed in embryonic cortices of dicer-deleted mice [36, 66–72, 75], strongly indicate a requisite role for miRNAs in the control of cell survival. However, given that cell survival, cell cycle control and differentiation are tightly linked process, it is difficult to identify the direct role of miRNAs in cell survival. This is a very difficult challenge that will require further studies.

### Convergent miRNA actions control specification of cortical cell subtypes

Recent studies indicate that convergent miRNA actions control the specification of cortical progenitors subtypes. In the developing neocortex, the BP can be visualized by the expression of Tbr2 (Eomes), a transcription factor that controls their generation from apical progenitors [147]. In two independent studies, it has recently been found that miRNA members of the miR-17-92 cluster are required to maintain pools of apical and BP through convergent repression of the tumor suppressor protein Pten, and of the transcription factor Tbr2 (Fig. 3). In one of these studies, conditional deletion of miR-17-92 subfamily genes in the developing neocortex was obtained by expression of Cre driven by the *Emx1* promoter. In the embryonic neocortex of conditional knockout mice for miR-17-92 subfamily, apical progenitor proliferation was reduced, and transition of apical progenitors to BP was enhanced [52]. This phenotype was due to the parallel action of miR-19a and miR-92 repressing the expression of Pten and Tbr2 proteins, respectively (Fig. 3). Repression of Tbr2 by miR-92 was also observed in a parallel study [77]. However, given that some miRNAs from the miR-17-92 subfamily share similar seed sequences, it is likely that some of those miRNAs also converge on the same targets. Taken together, these results indicate that convergent miRNA actions control NPC subtype specification in embryonic neocortex.

As introduced above, neurons derived from a common pool of progenitors adopt distinct laminar fates according to their birth order during cortical development. Conversely, cortical neurons that are born at the same time migrate in the same layer and share many morphological/functional properties, including layer-specific projection patterns [6, 148]. Therefore, the timing of progenitor division and the generation of different subtypes of cortical neurons are tightly regulated events [41]. Several years of studies have defined the temporally regulated expression of transcription factors that control some of these aspects in cortical development [6]. However, a critical question about this paradigm is how the timing of those transcription factors is regulated. The recent observation that dicer deletion results in the progressive restriction of RG competence [76] suggests that miRNAs also play an important role in this process.

In mammalian corticogenesis, the transcription factor Foxg1 regulates the competence of neural precursors by suppressing the generation of the earliest-born neurons, the Cajal-Retzius cells [149]. Intriguingly, the double knockout mouse for two of the genes encoding miR-9 and -9* (miR-9-2 and miR-9-3 double knockout mice, miR-9-2/3 KO) showed that this miRNA can either promote or suppress NSC/NPC proliferation through different targets at different stages of brain development [37]. At early developmental stages (i.e., E12-E13), miR-9 represses Foxg1 (Fig. 3) and by this means promotes the generation of Cajal-Retzius cells in the medial pallium of developing telencephalon [150, 151]. Conversely, miR-9-2/3 KO mice showed upregulation of Foxg1, as well as greatly reduced numbers of Cajal-Retzius cells and other early born neurons. Consistent with this finding, miR-9 overexpression in the VZ at E13.5 causes precocious neuronal differentiation [109], and miR-9 knockdown in mouse embryonic stem cells increases the number of glial cells at the expense of neuron production [128]. At later developmental stages (i.e., from E15. 5 to E18. 5), NPC proliferation in the dorsal telencephalon of miR-9-2/3 double knockout mice was significantly suppressed, and this correlated with elevated expression of TLX [37]. The regulation of TLX by miR-9 at E15. 5-E18. 5, but not at E13. 5, seems to result from a difference in the cellular context (Fig. 3). The diverse functions of miR-9 at different stages of development, and thus in different cellular contexts, provide a further level of complexity to the miRNA-mediated control of cortical development.

The implication of miRNAs in the specification of additional subtypes of cortical neurons is largely unknown. However, recently it has been shown that miR-125b and miR-181a specifically promote the generation of dopaminergic neurons, whereas miR-181a* inhibits the development of this neuronal subtype (Fig. 3) [152]. Another study characterized miRNA profiles in glutamatergic and GABAergic neurons, and found several miRNAs that are selectively enriched in each of these neuronal subtypes [53].

These studies are the first steps toward the investigation of the role of miRNAs in the control of the specification of cortical cell subtypes.

### Convergent miRNA actions control neurite outgrowth and migration in developing glutamatergic cortical neurons

Radial migration of cortical neurons requires precise coordination of leading process extension and branching [153–157]. Interestingly, dicer deletion, or manipulation of specific miRNAs, such as miR-34, miR-124, miR-9, miR-132/212and miR-134, revealed that miRNAs control neurite outgrowth and elaboration in cultured neurons [74, 78, 158–164]. Direct evidence of miRNA-dependent function(s) in the control of radial migration of glutamatergic neurons is still scarce in developing neocortex; however, some evidence of convergent miRNA actions that might regulate neurite outgrowth and possibly neuronal migration has recently been published. One such example is provided by the convergence of miR-9 and miR-132 on their common target Foxp2 [74] (Fig. 3). Foxp2 is a member of the Fork head-box family of transcription factors involved in the fine control of neurite outgrowth in neurons during cortical development, and in the evolution of speech and language in humans [165]. The convergent action of miR-9 and miR-132 on Foxp2 has recently been shown to control outgrowth/elaboration of neurites in cultured neurons, and radial migration of glutamatergic cortical neurons in developing mouse neocortex [74]. Intriguingly, miR-9 was also found to ensure proper development and migration of motor neurons by tuning levels of Foxp1 in developing chick spinal cord [166]. Given that Foxp1 and Foxp2 are highly related genes that are both expressed in the mammalian embryonic cortex, and that miR-9 binding sites in both Foxp1 and Foxp2 are evolutionarily conserved [74, 166] (Fig. 3), it is possible to speculate that miR-9 might control radial migration in glutamatergic cortical neurons through a functional convergence on Foxp1 and Foxp2.

MiR-132 and miR-212 might provide another example of convergent miRNA actions to control radial migration of glutamatergic cortical neurons. These two miRNAs are generated by the processing of a common transcript, the pri-miR-132/212, which has been shown to give rise to four miRNA species; miR-132, miR-212 as well as miR-132* and miR-212*. miR-132 (the most-abundant miRNA produced by the pri-miR-132/212 transcript) is a neuronal-enriched miRNA that is rapidly enhanced by the cAMP response element-binding (CREB) protein [167], and is generally regarded as an activity-dependent miRNA that controls synaptogenesis and synaptic plasticity in the adult brain visual cortex and adult hippocampus [168–171]. Much of the initial work on miR-132/212 function has relied on overexpression, or inhibition by means of miRNA mimics or inhibitors, respectively. While this approach provides a powerful way to start to dissect the function of a specific miRNA, it is possible that it may give rise to non-physiological off-target effects. For example, in cortical neurons, in vitro overexpression of miR-132 induced neurite outgrowth; conversely, inhibition of miR-132 function attenuated neurite outgrowth, by targeting p250GAP, a GTPase-activating protein that inhibits the activities of Rac and Cdc42 (Fig. 3) [158]. Consistently, in vivo inhibition of miR-132/212 using electroporation of a miR-132/212 sponge led to reduced dendritic complexity and spine density, while overexpression had the opposite effects [172]. Moreover, knockout of miR-132/212 in vivo can affect the dendritic growth and arborization of newborn neurons in the dentate gyrus of the adult hippocampus, and these effects occurred, partially, through the actions of miR-132 on p250GAP [161]. However, a recent study has shown that loss of miR-132/212 in double knockout mice impairs synaptic function, but does not alter neuronal morphology, and indeed the levels of the previously reported miR-132 targets, p250GAP, MeCP2 and p300, were not significantly changed in miR132/212 double knockout mice (although the protein levels of p250GAP were not determined in this study) [173]. Thus, to date, the mechanism of miR-132/212-dependent control of neurite outgrowth, elaboration and synaptogenesis, and therefore migration, remains controversial.

Finally, miR-134 was recently implicated in the control of neurite outgrowth and neuronal migration. MiR-134, which in the adult brain is abundantly expressed in glutamatergic neurons and controls spine development and homeostatic plasticity [174], has been found to exert stage-specific effects on cortical progenitors, migratory neurons and differentiated neurons during cortical development. In particular, miR-134 has been shown to regulate Doublecortin (Dcx) and Chrdl-1, a BMP antagonist (Fig. 3) [163]. Moreover, in NPCs, miR-134 promotes cell proliferation and counteracts Chrdl-1-induced apoptosis and Dcx-induced differentiation. Whereas, in differentiating neurons miR-134 modulates process outgrowth in response to exogenous BMP-4 in a noggin-reversible manner, and reduces cell migration in vitro and in vivo in a Dcx-dependent manner [163]. We speculate that these parallel actions of miR-134 might represent another example of convergent miRNA actions to control maturation, and possibly migration of glutamatergic neurons. However, further studies will be needed to clarify this aspect in detail.

In short, despite the evidence indicating that convergent miRNA actions might control neurite extension and branching, the picture of miRNA-dependent regulation of cortical neuron migration still remains a poorly characterized aspect of miRNA-dependent control of cortical development.

## Conclusions

The development of the mammalian neocortex requires the precise orchestration of intrinsic signaling pathways and extrinsic factors that govern the time of appearance, the relative proportions and the position of cortical cell types, as well as the formation of functional neuronal networks. These events need to be finely regulated at the molecular level, and miRNAs are particularly well suited to exert such a broad regulatory function. Indeed, a single miRNA has the potential to target a large number of genes in parallel [101, 102], most of the mammalian genes are putative targets of miRNAs [12], and accumulating evidence indicates that co-expressed miRNAs can simultaneously modulate genes with redundant functions, thereby achieving a greater effect on important aspects of cortical development.

In this review, we have collected and critically evaluated all the evidence about in-vivo-validated miRNAs–target interaction with relevance to neocortical development, and proposed the model of convergent miRNA actions. Our model not only encompasses the concept of miRNA cooperativity recently proposed by Schouten and colleagues [18], but also appears to be consistent with the previously proposed “tuning” function of animal miRNAs [175], and therefore might explain a possible mechanism used by miRNA to control cortical development. In contrast, our model seems incompatible with the provocative hypothesis proposed by Seitz [176] that miRNAs cannot fine-tune the transcriptome by targeting many mRNAs at the same time, but rather most of the putative targets act as “decoys” or “false targets” to sequester miRNAs and inhibit their function toward the authentic and functionally relevant targets. While, Seitz’s hypothesis might still be valid for specific miRNAs (e.g., developmental switches such as lin-4 and let-7) and animal species (e.g., *C.* *Elegans*), it is now apparent that the complexity of the cellular environment (genetic and epigenetic status, sequence, expression levels and stoichiometry of direct targets and miRNPs, etc.) also contributes to miRNA functions. We therefore speculate that the repression of the supposed “decoys” might be relevant in other cellular contexts, or for a different function of miRNAs.

In fact, by converging on a common target, multiple miRNAs might compensate the mild degree of mRNA regulation that is mediated by a single miRNA, and this is especially important when the degree of binding of the miRNA to its target is weak [94–96]. On the other hand, a single miRNA, despite its mild degree of mRNA regulation, might still achieve a meaningful biological effect by converging on different genes in the same pathway, or exerting redundant functions. Thus, the convergent miRNA actions might be particularly important for a dynamic system such as the developing neocortex. For example, convergent miRNA actions might contribute to silence functions that are no longer important, or are dangerous (e.g., oncogenes), and at the same time shift the proteome toward a new equilibrium in other cells (e.g., committed cells). Consistent with the latter scenario, several studies point out that miRNA functions are particularly required for cell fate transitions in developing neocortex [36, 68, 69, 71, 73, 75, 76, 81]. It should also be mentioned that in most cases, we found examples of two miRNAs that converge on a single target gene. This result might give the false impression that convergent miRNA actions mostly involve only two miRNAs, an implication that seems to contradict our definition of convergent miRNA actions. However, it is possible that this result is due to our current lack of knowledge about miRNA-target interactions. In fact, evidence of three miRNAs targeting a single gene exists in the literature (e.g., Krek et al. [177]), but at present this evidence is still scarce and most of these data have not been validated in vivo. Further functional studies will certainly clarify this aspect in the near future.

Last, but not least, convergent miRNA actions, and the crosstalk with additional noncoding RNAs might have exerted an important role in the evolution of the human brain. This seems to contrast with the prevailing knowledge that several miRNAs and their binding sites in target genes are often evolutionarily conserved among distant species [11, 12]. However, given our current lack of knowledge of miRNA functions in developing chordates, it is possible to speculate that some of the evolutionarily conserved miRNA-target interactions might control basic aspects of corticogenesis (e.g., neurogenesis or gliogenesis), while some of the convergent miRNA actions might not be conserved, thus providing an evolutionary advantage (e.g., increasing the number of asymmetric divisions in NSCs/NPCs, or increasing the numbers of oRG cells, thus increasing the numbers of neurons generated during corticogenesis). Given the 47:1 ratio of transcribed noncoding regions to coding regions in humans, compared to the 43:1 ratio in mice and to the 2.4:1 ratio in *Drosophila*, it is now beyond question that the importance of the noncoding part of the genome has grown in parallel with evolution [178].

In short, we predict that the coming years will witness an avalanche of studies demonstrating a prominent role for convergent actions of miRNAs (including the many non-canonical, or atypical miRNA-like species) that control development and evolution of the neocortex.
